# Using perceptive computing in multiple sclerosis - the Short Maximum Speed Walk test

**DOI:** 10.1186/1743-0003-11-89

**Published:** 2014-05-27

**Authors:** Janina Behrens, Caspar Pfüller, Sebastian Mansow-Model, Karen Otte, Friedemann Paul, Alexander U Brandt

**Affiliations:** 1 NeuroCure Clinical Research Center, Charité - Universitätsmedizin Berlin, Charitéplatz 1, 10117 Berlin, Germany; 2 Motognosis UG (haftungsbeschränkt), Berlin, Germany; 3 Clinical and Experimental Multiple Sclerosis Research Center, Department of Neurology, Charité - Universitätsmedizin Berlin, Charitéplatz 1, 10117 Berlin, Germany

**Keywords:** Computerized gait assessment, Gait impairment, Multiple sclerosis, Walking speed, T25FW

## Abstract

**Background:**

We investigated the applicability and feasibility of perceptive computing assisted gait analysis in multiple sclerosis (MS) patients using Microsoft Kinect™. To detect the maximum walking speed and the degree of spatial sway, we established a computerized and observer-independent measure, which we named Short Maximum Speed Walk (SMSW), and compared it to established clinical measures of gait disability in MS, namely the Expanded Disability Status Scale (EDSS) and the Timed 25-Foot Walk (T25FW).

**Methods:**

Cross-sectional study of 22 MS patients (age mean ± SD 43 ± 9 years, 13 female) and 22 age and gender matched healthy control subjects (HC) (age 37 ± 11 years, 13 female). The disability level of each MS patient was graded using the EDSS (median 3.0, range 0.0-6.0). All subjects then performed the SMSW and the Timed 25-Foot Walk (T25FW). The SMSW comprised five gait parameters, which together assessed average walking speed and gait stability in different dimensions (left/right, up/down and 3D deviation).

**Results:**

SMSW average walking speed was slower in MS patients (1.6 ± 0.3 m/sec) than in HC (1.8 ± 0.4 m/sec) (p = 0.005) and correlated well with EDSS (Spearman’s Rho 0.676, p < 0.001). Furthermore, SMSW revealed higher left/right deviation in MS patients compared to HC. SMSW showed high recognition quality and retest-reliability (covariance 0.13 m/sec, ICC 0.965, p < 0.001). There was a significant correlation between SMSW average walking speed and T25FW (Pearson’s R = -0.447, p = 0.042).

**Conclusion:**

Our data suggest that ambulation tests using Microsoft Kinect™ are feasible, well tolerated and can detect clinical gait disturbances in patients with MS. The retest-reliability was on par with the T25FW.

## Background

Multiple sclerosis (MS) is a common chronic inflammatory and neurodegenerative disease that normally begins in young adulthood, typically affecting patient quality of life and leading to high rates of early retirement [1-4]. Impairment of gait and balance are major factors that restrict daily activity [5] and may occur as early as after a first clinical episode [6-8].

In current clinical practice, balance and gait impairment are quantified using a combination of clinical examination and patients’ reported maximum walking distance: The Kurtzke‘s Functional Systems and Expanded Disability Status Scale (EDSS) [9] are widely used both in clinical practice and for clinical trials. The EDSS provides a good overview of current neurologic status, gait impairment and mobility dysfunction, but it has limitations. It is a relatively subjective measure with high intra- and inter-rater variability and quantifying mild symptoms and symptom progression is challenging [10,11]. The Timed 25-Foot Walk (T25FW) measures the time a patient takes to walk 25 feet at maximum speed. As part of the Multiple Sclerosis Functional Composite (MSFC), the T25FW is currently the most widely implemented method to objectively quantify gait disability in clinical MS trials and, to a lesser extent, clinical practice [12,13]. Although an excellent method of quantifying overall gait disability, the T25FW measures only the time taken to walk a set distance [14]. As alternatives to the T25FW, timed walking tests might improve reliability. In particular, the 2-minute walk test is proposed as an additional outcome parameter in clinical trials [15].

Recently, more objective motion capture systems (for an overview see [16]) have been proposed as tools for detecting the pattern of gait impairment more precisely than clinical examination using conventional tools (i.e. EDSS) [17,18]. However, none of these systems has found its way into clinical routine yet, and are therefore rarely available in outpatient clinics and neurologic practices.

With an infrared light camera system, the Microsoft Kinect™, which was originally developed for video gaming, detects anatomical landmark positions in three dimensions (3D) [19]. This function is based on a decision forest method that harnesses mass data of sensor-recorded skeletal joint movements [20]. Using the programming interface made available via the Kinect software development kit (SDK) [21], it has in the past been employed to detect balance and motion of healthy subjects [22,23] and stroke patients [24,25]. Furthermore, some few studies have reported methods for gait parameter approximation using Kinect [26-28], however, none of these has been applied to patients with MS.

The objective of this study was to evaluate the feasibility of computerized versions of walking tests using Microsoft Kinect and to compare the resulting findings for MS patients with low to moderate neurological impairment to those for healthy controls. We established the Short Maximum Speed Walk test (SMSW) as a measure to analyse patients’ gait speed and degree of sway. We report feasibility, reliability and correlation of results with those of EDSS and the T25FW.

## Methods

### Ethics

The local ethics committee of the Charité – Universitätsmedizin Berlin approved the study (EA1/225/12). It was conducted in accordance to the Declaration of Helsinki in its currently applicable version. All patients and healthy subjects gave written informed consent.

### Patients

Twenty-two patients with diagnosed MS according to the current panel criteria [29] (age mean ± SD 43 ± 9 years, 13 female) and 22 age and sex matched healthy controls (HC) (age 37 ± 11 years, 13 female) were enrolled in the study. MS patients were recruited from on-going clinical trials at Charité’s neuroimmunology outpatient clinic. HC were recruited from volunteers. Blinding was not attempted, since the operators knew many subjects and visual contact was mandatory. MS patients were first clinically graded using EDSS (median 3.0, range 0.0 – 6.0) under supervision of a neurologist. The EDSS is calculated from a detailed neurological examination using functional system scores that assess the visual system function, brainstem function, pyramidal tract function, cerebellar function, sensory system function, bowel and bladder function, cerebral function and patient ambulation [9]. Subjects additionally performed the T25FW component of the Multiple Sclerosis Functional Composite (MSFC) [30]. T25FW results were not available from eight of the 22 HC, and one of the 22 MS patients. The first 8 HC had been tested before the T25FW was added to the study protocol and the MS patient could not complete the study in full due to time constraints.

### Perceptive computing assisted motor assessment

**Figure 1:**
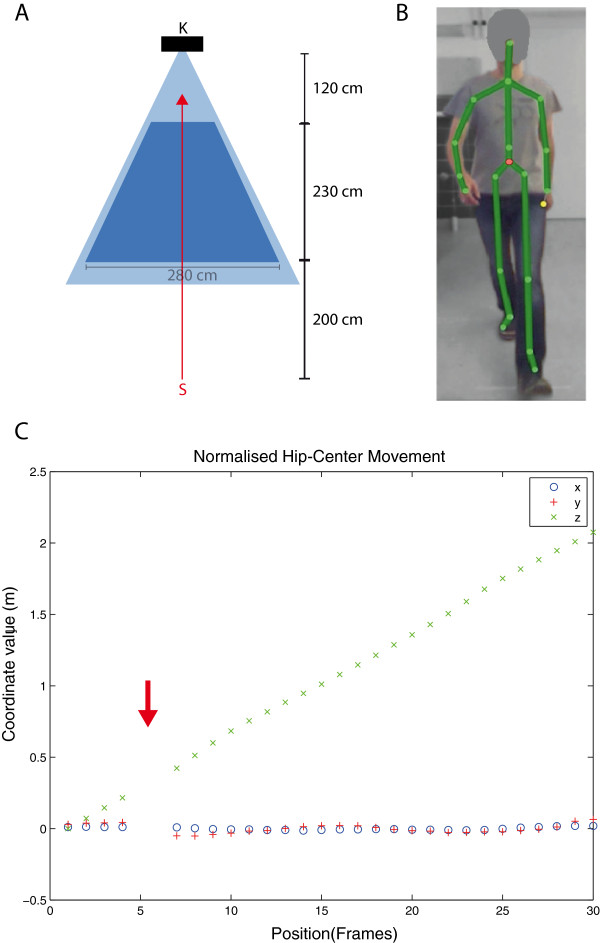
**System setup. A)** Schematic system setup (view from above): The Kinect sensor (K) was positioned 140 cm above ground, angled -9° towards the floor. Starting (S) about 2 metres in front of the beam path (light blue area), subjects walked with maximum speed towards the Kinect camera (red arrow). Recording started and stopped automatically as sensors detected the subjects entering and leaving the detection zone (dark blue area). **B)** Sample screenshot of a healthy subject during the test with skeleton projection (green lines). The *hip-centre* joint (marked as red dot) was used as the data source for analysis. **C)** Sample data from single healthy subject after normalization. At the position of the red arrow a measurement frame was removed due to a detected calibration jump.

We used custom-built software running on a Windows 8 (Microsoft Corp., Redmond, WA, USA) computer with Kinect Software Development Kit (SDK) version 1.7. A Microsoft Kinect sensor was attached to a fixed pole, thereby covering a triangular area of roughly 2.5×2.5 m. The Kinect system records live videos with a conventional camera and combines these with depth information comprising a combined feed from an infrared projector and an infrared camera. The Kinect Software Development Kit (SDK) then detects the human subject in the 3D video in real-time and models an artificial skeleton with 20 joints of an individual subject and their movement over time [19,21]. A sample of the process is shown in Figure 1 and Supplementary Video 1. Each subject was tested in an evenly lit room in a single session of sequential tests. All subjects were given the same instruction as specified in a standardized test procedure: “Walk as fast as you can towards the sensor”. The starting point for walking was approximately two metres outside the detection range of the sensor. We postulated that this would allow the subject to reach maximum walking speed before reaching the measurement zone. The start was given as a voice command. An automatic computer-generated sound signalled the subject to end the experiment after leaving the opposite edge of the sensor measurement zone.

### Short Maximum Speed Walk test analysis

The SDK’s recorded skeletal information was post-processed at a frame-rate of 30/s. To assess walking speed and related parameters we extracted the hip-centre joint coordinates over time. An overview of all parameters is given in Table 1. In detail, the data post-processing and analysis comprised the following steps:

**Table 1:** Overview of SMSW parameters

**Parameter**	**Unit**	**Description**	**Interpretation**
**Average walking speed**	m/s	Average speed during the measurement calculated as observed distance divided by observed time	Main outcome parameter; measures a subject’s average walking speed during the test
**Speed deviation**	m/frame	Standard deviation of speed between sequent frames	Measure of directional speed homogeneity
**3D direction deviation**	m^2^	Mean square error from the Euclidean distance between the actual position and the position from a linear model approximation over the main z-vector	Combined expression of how much directional, up/down and left/right variability a subject’s gait shows
**Left/right deviation**	cm	Left/right deviation between the actual position and the position as determined by a linear model approximation over the main z-vector	Parameter describes a subject’s left/right movement during the walking test.
**Up/down deviation**	cm	Up/down deviation between the actual hip center position and the position from a linear model over the main z-vector.	Parameter describes a subject’s up/down movement during the walking test.
Observed time	s	Total time the subject was tracked	Quality parameter
Observed distance	m	Total distance the subject was tracked	Quality parameter
Jump count		Number of detected calibration jumps during the measurement	Quality parameter
Jump distance	m	Mean distance of all calibration jumps detected during the measurement	Quality parameter
Measurement length	frames	Total number of frames collected	Quality parameter

Readout parameters are given in bold; quality parameters are shown in regular font.

First, joint coordinates were normalized, correcting for sensor tilt and walking direction of the subject by computing a linear approximation of the hip-centre movement, translating the start of the movement to the point of origin and rotating the direction of movement onto the positive Z-axis.

As in any automated assessment tool, artefacts (in the form of tracking errors, or calibration jumps) were inevitable. Specifically, detection of the spatial location of the crucial *hip-centre* joint by the SDK system was clearly sometimes erroneous as determined by visual inspection. Furthermore, the SDK’s in-built error detection system (SDK Recognition Quality) was unable to identify these artefacts with sufficient accuracy, and instead falsely reported all joint detections as successful. To address this problem, we developed an alternative error correction technique. Here, we mathematically extracted the acceleration of the joint movement between two frames as recorded by the SDK system. We analysed the transition between the points in terms of a subject’s overall acceleration and averaged these values across the study cohort. We found that if aggregated acceleration over all coordinate axes exceeded a threshold of 0.6 m/frames^2^, the frames were likely to be an artefact. As such they were flagged as suspected tracking errors (“calibration jump”) and excluded from further analysis.

Using the calibration jump-corrected data, we computed the SMSW test parameters given in Table 1, which were then used for statistical analysis.

All data post-processing and analysis was performed using Matlab 2012A (Mathworks, Ismaning, Germany).

### Analysis of measurement quality

We derived several quality parameters and investigated the recognition quality of the central joint *hip-centre* (Table 2). The frequency of calibration jumps was comparable between HC and MS patients. However, the average jump size was significantly higher in HC than MS patients. There was a strong correlation between walking speed and average jump distance (Pearson’s R = 0.778, p < 0.001), suggesting that the reason for this difference was the higher speed of HC ambulation. Furthermore, the faster people were walking, the shorter were both the observed time (Pearson’s R = -0.967, p < 0.001) and the observed distance (Spearman’s Rho = -0.686, p < 0.001). This was probably caused by the short initialisation period the system needed when a subject walked into the detection range. As a result, MS patients were measured over a minimally longer time period and distance than HC, although the predefined test criteria were identical (Table 2).

**Table 2:** Quality parameters

	**HC**				**MS**				**t test**		
**Description**	**Mean**	**SD**	**Min**	**Max**	**Mean**	**SD**	**Min**	**Max**	**Mean Δ**	**SD Δ**	***P***
Observed time (sec)	1.232	0.399	0.780	2.636	1.535	0.363	0.998	2.376	0.303	0.115	0.012
Observed distance (m)	2.150	0.087	1.993	2.423	2.283	0.089	2.098	2.443	0.134	0.027	<0.001
Number of calibration jumps	0.9	1.0	0.0	3.3	0.9	1.3	0.0	5.3	-0.05	0.35	0.897
Average calibration jump distance (m)	0.091	0.033	0.044	0.168	0.058	0.013	0.029	0.074	-0.033	0.009	0.002
Measurement length (frames)	37.3	12.2	24.3	79.7	46.8	10.9	30.7	72.0	9.5	3.5	0.009

A detailed description of investigated parameters is given in Table 1.

*Abbreviations:HC* healthy controls, *MS* multiple sclerosis patients, *SD* standard deviation.

### Analysis of immediate retest reliability

T25FW tests were performed twice and the SMSW three times in a row to assess the retest reliability. T25FW, average speed and SMSW showed excellent retest reliability with ICC > 0.9 in HC and MS. Speed deviation and up/down deviation showed good reliability, whereas reliability for 3D direction deviation and left/right deviation was only moderate (for details, see Table 3). When data was not corrected for calibration jumps, ICC was considerably lower (not shown).

**Table 3:** Immediate retest reliability

	**HC**			**MS**		
**Description**	**ICC**	**CI LOW**	**CI UP**	**ICC**	**CI LOW**	**CI UP**
T25FW*	0.905	0.714	0.969	0.990	0.976	0.996
SMSW Average speed	0.989	0.979	0.995	0.980	0.960	0.991
SMSW Speed deviation	0.742	0.480	0.884	0.901	0.800	0.955
SMSW 3D direction deviation	0.429	-0.152	0.744	0.540	0.072	0.794
SMSW Left/right deviation	0.533	0.058	0.790	0.596	0.186	0.819
SMSW Up/down deviation	0.958	0.916	0.981	0.936	0.871	0.971

*) The T25FW analysis is based on data from 21 MS patients and 14 HC.

*Abbreviations:* HC, healthy controls; MS, multiple sclerosis patients; SD, standard deviation; T25FW, Timed 25-Foot Walk; ICC, 1-way Intra-class correlation coefficient; CI, confidence interval; LOW, lower bound; UP, upper bound.

### Statistical analysis

Immediate retest reliability was analysed using intra-class correlation coefficients (ICC) with a one-sided, random model. Test results between MS patients and HC were compared using t-tests. When equal variances could be assumed (Levene’s test p > 0.05), p-values from independent sample Student’s t-tests are given. When equal variances could not be assumed (Levene’s test p < 0.05), results from the Welch’s t-test are given. The association between T25FW and SMSW measures was investigated using Pearson’s correlation analysis and a Bland-Altman analysis. Correlation with EDSS and functional system scores was investigated with Spearman’s Rho analysis due to the ordinal nature of EDSS data.

Statistical analysis was performed using SPSS version 21 (IBM, Armonk, NY, USA). A p < 0.05 was deemed significant. All tests should be understood as exploratory data analysis as no prior power calculation and subsequent corrections for multiple testing were applied.

## Results

**Figure 2:**
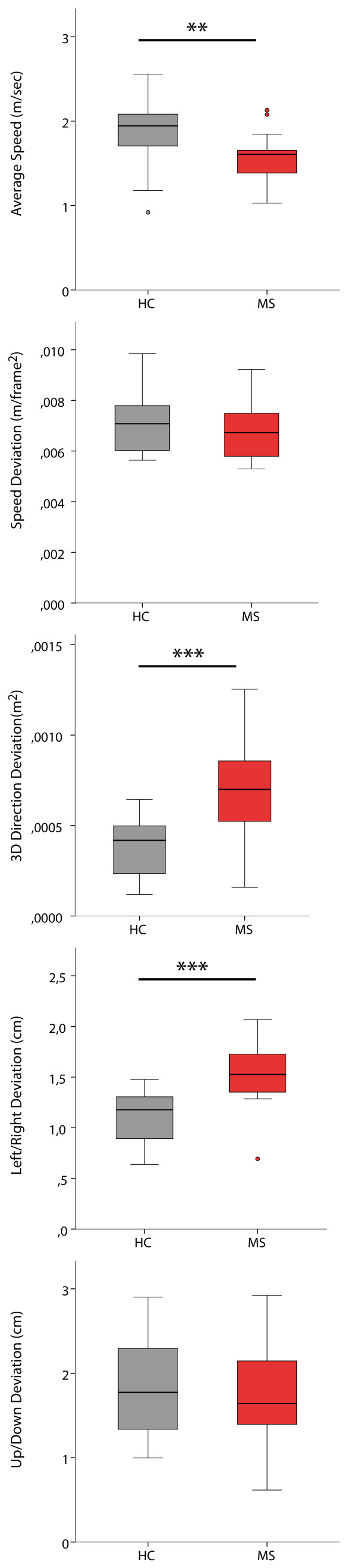
**Test outcome differences.** Box plots of test outcome measurements (red = MS patients, grey = healthy subjects). Significance levels from t-tests: *** = p < 0.001; ** = p < 0.01; * = p < 0.05.

Five gait parameters were derived from the hip-centre joint movement during the walk test and compared between HC and MS patients. Average walking speed was the main test parameter, while the four other parameters described gait stability in different dimensions (Table 1). MS patients were significantly slower than HC in average walking speed. In MS patients, 3D direction deviation and left/right deviation were significantly higher than in HC, whereas there was no significant difference between speed deviation and up/down deviation (Table 4 and Figure 2).

**Table 4:** Comparison between healthy controls and multiple sclerosis patients

**Parameter**	**HC**				**MS**				**t test**		
	**Mean**	**SD**	**Min**	**Max**	**Mean**	**SD**	**Min**	**Max**	**Mean Δ**	**SD Δ**	***P***
Average speed (m/s)	1.852	0.366	0.919	2.557	1.555	0.294	1.029	2.131	-0.297	0.100	0.005
Speed deviation (m/frame^2^)	0.0070	0.0010	0.0056	0.0099	0.0068	0.0011	0.0053	0.0092	0.000	0.000	0.437
3D direction deviation (m^2^)	0.00040	0.00015	0.00012	0.00064	0.00072	0.00026	0.00016	0.00125	0.00032	0.00007	<0.001
Left/right deviation (cm)	1.122	0.243	0.638	1.477	1.537	0.301	0.693	2.069	0.415	0.082	<0.001
Up/down deviation (cm)	1.818	0.596	0.997	2.902	1.777	0.579	0.617	2.924	-0.041	0.177	0.817

See Table 1 for a detailed description of the parameters assessed.

*Abbreviations:HC* healthy controls, *MS* multiple sclerosis patients, *SD* standard deviation.

### Comparison between SMSW and T25FW

**Figure 3:**
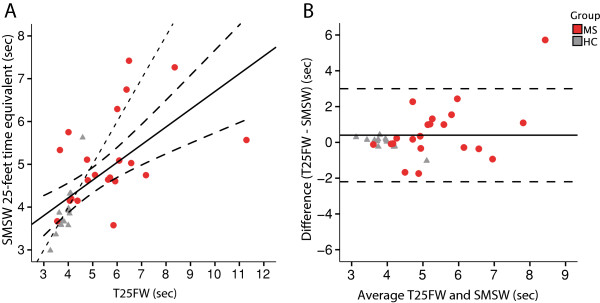
**SMSW in comparison with the T25FW.** Measuring agreement of gait speed detection of MS patients (red dots) and healthy subjects (grey triangles) with standard T25FW and SMSW. To be able to directly compare SMSW and T25FW in a Bland-Altman analysis, the SMSW time equivalent of walking 25-feet at the detected average speed was calculated. **A)** Results are plotted against each other. The solid line represents an R^2^ of 0.392 from a linear regression analysis; the long dashed lines are 95% confidence intervals to the mean. The short-dashed line represents the theoretical test equality (T25FW = SMSW). B) Bland-Altman plot comparison between T25FW and SMSW average speed. **B)** The difference of the mean is -0.4 sec (solid line). Long dashed lines are 2x standard deviation.

There was a significant but only moderate correlation between T25FW and SMSW average walking speed (Pearson’s R = -0.447, p = 0.042) in MS patients (Figure 3). In a Bland-Altman analysis comparing T25FW with SMSW and including HC and MS patients, the difference between the measures was 0.4 ± 1.3 sec (mean ± SD) (Figure 3). There was no correlation between the test difference and observed time, meaning that these differences could not be explained by a higher estimation error in tests with shorter measurement times.

### Association with EDSS and functional system scores

Both T25FW and SMSW correlated equally well with EDSS total score (Table 5A) and EDSS ambulation score (Table 5C). In the EDSS functional system scores (Table 5B), T25FW correlated mainly with visual function, cerebral function but not with pyramidal function. In contrast, SMSW did not correlate with visual function but with brainstem function, pyramidal and cerebral function (Table 5). Gait stability as expressed by the four deviation parameters correlated to a lesser extent with EDSS (Table 5).

**Table 5:** Correlation between test outcomes and EDSS

	**A**		**B**							
**Parameter**	**Total score**		**FS**	**VIS**	**BS**	**PYR**	**cer**	**SENS**	**B&B**	**CER**
	**Median**	3.0	**0**	12	7	3	4	2	9	5
	**Min**	0.0	**1**	9	10	6	8	8	7	9
	**Max**	6.0	**2**	1	5	8	6	10	5	8
			**3+**			5	4	2	1	
T25FW*	**Rho**	**0.694**	**Rho**	**0.492**	0.304	0.161	0.394	-0.081	0.397	**0.539**
	***P***	*<0.001*	***P***	*0.023*	*0.180*	*0.485*	*0.077*	*0.727*	*0.075*	*0.012*
SMSW Average speed	**Rho**	**-0.701**	**Rho**	0.060	**-0.516**	**-0.634**	-0.355	-0.126	-0.204	-0.430
	***P***	*<0.001*	***P***	*0.789*	*0.014*	*0.002*	*0.105*	*0.578*	*0.362*	*0.046*
SMSW Speed deviation	**Rho**	0.276	**Rho**	0.286	0.369	0.024	0.122	**-0.453**	0.155	0.291
	***P***	*0.213*	***P***	*0.198*	*0.091*	*0.917*	*0.588*	*0.034*	*0.491*	*0.189*
SMSW Derived T25FW	**Rho**	**0.676**	**Rho**	-0.060	**0.516**	**0.639**	0.327	0.090	0.204	**0.428**
	***P***	*0.001*	***P***	*0.789*	*0.014*	*0.001*	*0.137*	*0.690*	*0.362*	*0.047*
SMSW 3D direction deviation	**Rho**	**0.439**	**Rho**	0.086	0.411	0.196	0.074	0.048	-0.099	0.317
	***P***	*0.041*	***P***	*0.705*	*0.057*	*0.382*	*0.743*	*0.834*	*0.662*	*0.151*
SMSW Left/right deviation	**Rho**	**0.429**	**Rho**	0.072	0.392	0.191	0.033	0.007	-0.125	0.317
	***P***	*0.046*	***P***	*0.750*	*0.071*	*0.395*	*0.884*	*0.974*	*0.579*	*0.151*
SMSW Up/down deviation	**Rho**	-0.134	**Rho**	-0.122	-0.301	-0.181	-0.264	-0.246	0.056	-0.397
	***P***	*0.551*	***P***	*0.590*	*0.173*	*0.421*	*0.236*	*0.269*	*0.806*	*0.067*

P values are given in italic; correlation coefficients from tests with significant results are given in bold. A) Overview of EDSS distribution and results from a Spearman’s Rho analysis compared to the results of investigated measures and standard T25FW results. B) Detailed overview of FS distribution and Spearman’s Rho analysis results. *) The T25FW analysis is based on data from 21 MS patients and 14 HC.

*Abbreviations:T25FW* Timed 25-Foot Walk, *EDSS* Expanded Disability Status Scale, *FS EDSS* functional system score, *VIS* visual system score, *BS* brainstem score, *PYR* pyramidal tract score, *cer* cerebellar score, *SENS* sensory system score, *B&B* bowel and bladder score, *CER* cerebral score.

## Discussion

In this cross-sectional study we investigated the applicability and feasibility of perceptive computing assisted motion analysis in MS patients using the Microsoft Kinect system. Our primary question was whether skeletal tracking data recorded by the device’s SDK program would yield reliable information for gait analysis. Using only the *hip-centre* joint, we established the test SMSW to analyse MS patients’ gait at maximum walking speed. Additionally, we analysed the SMSW left/right and up/down deviation of MS patients gait in comparison to HC.

Using the SMSW, we were able to detect differences in average walking speed between MS patients and HC. Several gait stability parameters also showed promising results: Especially 3D direction deviation and left/right deviation showed prominent differences between HC and MS patients. The lateral sway of MS patients (described by the parameter left/right deviation) detected by our test suggests that stability impairments are indeed detectable by perceptive computing systems, even over limited observation time and distance (although some types of gait variability may only become evident over longer distances) [31].

The linear distance covered by the recognition area was only 2.2 metres (see Figure 1 and Table 2), and consequently only a few steps of each subject were analysed. Despite this short walking and recording time, the overall detection quality of the target *hip-centre* joint of the SMSW over time was excellent. Calibration jumps (i.e. tracking errors by the SDK system) presented regularly (see Table 2), however, these could be filtered out by applying a custom-built filter developed during the analysis of this experiment. After filtering out the calibration jumps, the retest reliability of the SMSW average walking speed parameter was excellent and on par with T25FW. The gait stability parameters showed less retest reliability. This was likely due to the fact that most differences between MS patients and HC were only marginally significant. The reliability of the gait stability measurements was superior in MS patients, where a higher signal-to-noise ratio was expected due to weaker gait stability. Additionally, the detection limit was heavily influenced by the joint recognition quality of the sensor. In fact, one measure (3D direction deviation), originally designed to detect changes in walking stability, proved to be very sensitive to calibration jumps in walking direction, which primarily occurred at faster walking speed.

In combination, these data suggest that an assessment employing gait analysis at lower walking speed might improve detection of gait variability and stability in mobility impaired patients. This would not only lower detection errors in the form of calibration jumps but would also allow the system to record several step cycles for a more robust analysis. This is supported by a previous study investigating a Kinect-based system for gait measurements using 3D point cloud information instead of skeletal tracking data, which reported accurate estimation of gait parameters compared to a Vicon motion analysis system in healthy volunteers [32]. A second study used skeletal data from all reported joints to analyse a postural control assessment battery in healthy subjects [23]. Our study adds to these findings and shows that Kinect-based motion analysis is also feasible in MS patients and is able to reliably detect gait differences in comparison to healthy controls. Although the experimental setups and analysis approaches differ, all three studies show that inexpensive 3D perceptual computing can be reliably used to quantify motion impairment. Furthermore, a recent work of Stone and Skubic showed, Kinect can also be installed in the patient’s home as a means of frequently and conveniently assessing gait [28].

We expected that SMSW average speed and the T25FW would yield similar results. However, MS patients’ results sometimes differed strongly in the two tests. Although typical MS symptoms like reduced muscular strength, spasticity or balance deficiency are widely known to fluctuate [33], it is unlikely that random irregularities or gait variability are the key factors in this discrepancy, because retest reliability was high in both tests.

Instead, a different influence of functional impairments on both tests seems possible. SMSW and T25FW correlated well with overall EDSS degree of neurological disability. However, when drilling-down by comparing individual parameter results to the corresponding EDSS functional system scores (FS), we were surprised to find a difference between the SMSW and the T25FW: Whereas SMSW average walking speed showed a moderate correlation to cerebral function, T25FW results correlated with visual function, and SMSW walking speed showed correlation with the pyramidal scores of the EDSS.

Pyramidal impairment such as muscular weakness and spasticity clearly restricts the gait of many MS patients [34,35]. However, as stated above, we found no correlation between the T25FW and the pyramidal function score of the EDSS. This is in discrepancy with previous findings, such as those of Phan-Ba et al. [36], and likely explained by the only mild pyramidal impairment of many patients in our study (41% patients with FS ≤ 2 ). In contrast to T25FW, SMSW correlated with pyramidal functional system scores. This might be explained by findings from previous studies where MS patients, performing the T25FW, accelerated walking speed after a relatively slow start [36,37]. Because muscular rigidity is triggered by postural changes [38], such as starting from a static position to walk, we may have detected a starting weakness of spastic patients. Thus, a patient, who begins moving more slowly, might show a slower walking time at SMSW average walking speed compared to T25FW. In the T25FW a slower speed at start might level over the longer distance. In this regard SMSW average walking speed may be more sensitive to impaired initiation of movement in MS patients than the T25FW. We did not evaluate spasticity with a validated assessment, so we were not able to further pursue the question of whether the SMSW could be a marker for minor pyramidal weakness.

An exploratory pilot study of this type has some limitations. Only Kinect-derived skeletal tracking information generated by the SDK was used [19]. Although not within the scope of the present study, it is conceivable that exclusive or hybrid inclusion of raw sensor data might yield additional or better results. Furthermore, we neither selected MS patients with specific and well-established motor symptoms or gait disability, nor did we compare SMSW test results to objective gait measures other than T25FW. The analysis between SMSW, T25FW and EDSS functional system scores should be treated as preliminary and the results should be interpreted with care. However, the demonstrated correlations fit well within the expected clinical framework and the results of previous studies. As such, our results can be expected to serve as a basis for confirmatory studies. Most importantly, the ability of SMSW measurements to detect changes longitudinally and the retest reliability of the test needs to be validated in a follow-up study.

## Conclusion

Perceptive computing-based detection of ambulation speed via the joint *hip-centre* was feasible and reliable. SMSW average walking speed was a valid parameter as demonstrated by retest reliability results and the strong correlation with established clinical scores, such as the T25FW and EDSS. A notable difference was the slower walking speed of MS patients measured using SMSW (mean distance: 2.2 metres) compared to T25FW (mean distance: 7.6 metres). The gait stability parameters showed greater left/right deviation in MS patients compared to HC. The analysis approach presented here shows promise as an objective technique for detecting and assessing of gait pattern and as a simple and affordable tool in the clinician’s toolbox. Finally, this study provides evidence that further investigation of gait with periodic Kinect-measurements may give new insight into the disease progression of MS.

## Competing interests

This study was funded in part with a grant from Novartis Pharma Germany. Motognosis is a company that has a commercial interest in the results of this research and technology. AUB and SMM are co-founders and shareholders of Motognosis. AUB, SMM and KO receive monetary compensation as employees from Motognosis. JB, CP, KO and FP report no potential conflict of interest. FP and CP were supported by the German Research Council (DFG Exc 257).

## Authors’ contributions

AUB, CP and FP planned and designed the study. JB and CP were responsible for supervising subject recruitment and measurement. KO and SMM developed feature extraction and performed raw data analysis. JB and AUB performed the statistical analysis. JB and AUB wrote the manuscript. FP revised the manuscript. All authors provided key intellectual content during the study design or manuscript revision. All authors read and approved the final manuscript.

## References

[B1] Noseworthy JH Lucchinetti C Rodriguez M Weinshenker BG Multiple sclerosis N Engl J Med 2000 343 13 938 952 10.1056/NEJM200009283431307 11006371

[B2] Patwardhan MB Matchar DB Samsa GP McCrory DC Williams RG Li TT Cost of multiple sclerosis by level of disability: a review of literature Mult Scler 2005 11 2 232 239 10.1191/1352458505ms1137oa 15794399

[B3] Krause I Kern S Horntrich A Ziemssen T Employment status in multiple sclerosis: impact of disease-specific and non-disease-specific factors Mult Scler 2013 19 13 1792 1799 10.1177/1352458513485655 23635910

[B4] Borisow N Döring A Pfueller CF Paul F Dörr J Hellwig K Expert recommendations to personalization of medical approaches in treatment of multiple sclerosis: an overview of family planning and pregnancy EPMA J 2012 3 1 9 10.1186/1878-5085-3-9 22738272 PMC3464716

[B5] Findling O Sellner J Meier N Allum JHJ Vibert D Lienert C Mattle HP Trunk sway in mildly disabled multiple sclerosis patients with and without balance impairment Exp Brain Res 2011 213 4 363 370 10.1007/s00221-011-2795-8 21773798

[B6] Kalron A Achiron A Dvir Z Motor impairments at presentation of clinically isolated syndrome suggestive of multiple sclerosis: Characterization of different disease subtypes NeuroRehabilitation 2012 31 2 147 155 22951709 10.3233/NRE-2012-0784

[B7] Kalron A Achiron A Dvir Z Muscular and gait abnormalities in persons with early onset multiple sclerosis J Neurol Phys Ther 2011 35 4 164 169 10.1097/NPT.0b013e31823801f4 22052130

[B8] Martin CL Phillips BA Kilpatrick TJ Butzkueven H Tubridy N McDonald E Galea MP Gait and balance impairment in early multiple sclerosis in the absence of clinical disability Mult Scler 2006 12 5 620 628 10.1177/1352458506070658 17086909

[B9] Kurtzke JF Rating neurologic impairment in multiple sclerosis: an expanded disability status scale (EDSS) Neurology 1983 33 11 1444 1452 10.1212/WNL.33.11.1444 6685237

[B10] Noseworthy JH Vandervoort MK Wong CJ Ebers GC Interrater variability with the Expanded Disability Status Scale (EDSS) and Functional Systems (FS) in a multiple sclerosis clinical trial. The Canadian Cooperation MS Study Group Neurology 1990 40 6 971 975 10.1212/WNL.40.6.971 2189084

[B11] Goodkin DE EDSS reliability Neurology 1991 41 2 Part 1 332 332 1992392 10.1212/wnl.41.2_part_1.332

[B12] Fischer JS Rudick RA Cutter GR Reingold SC The Multiple Sclerosis Functional Composite Measure (MSFC): an integrated approach to MS clinical outcome assessment. National MS Society Clinical Outcomes Assessment Task Force Mult Scler 1999 5 4 244 250 10.1177/135245859900500409 10467383

[B13] Rosti-Otajärvi E Hämäläinen P Koivisto K Hokkanen L The reliability of the MSFC and its components Acta Neurol Scand 2008 117 6 421 427 18081910 10.1111/j.1600-0404.2007.00972.x

[B14] Hobart J Blight AR Goodman A Lynn F Putzki N Timed 25-foot walk: direct evidence that improving 20% or greater is clinically meaningful in MS Neurology 2013 80 16 1509 1517 10.1212/WNL.0b013e31828cf7f3 23535489

[B15] Gijbels D Dalgas U Romberg A de Groot V Bethoux F Vaney C Gebara B Medina CS Maamâgi H Rasova K de Noordhout BM Knuts K Feys P Which walking capacity tests to use in multiple sclerosis? A multicentre study providing the basis for a core set Mult Scler 2012 18 3 364 371 10.1177/1352458511420598 21952098

[B16] Pearson OR Busse ME van Deursen RWM Wiles CM Quantification of walking mobility in neurological disorders QJM 2004 97 8 463 475 15256604 10.1093/qjmed/hch084

[B17] Sosnoff JJ Weikert M Dlugonski D Smith DC Motl RW Quantifying gait impairment in multiple sclerosis using GAITRite technology Gait Posture 2011 34 1 145 147 10.1016/j.gaitpost.2011.03.020 21531562

[B18] Sosnoff JJ Sandroff BM Motl RW Quantifying gait abnormalities in persons with multiple sclerosis with minimal disability Gait Posture 2012 36 1 154 156 10.1016/j.gaitpost.2011.11.027 22424761

[B19] Criminisi A Shotton J Konukoglu E Decision Forests for Classification, Regression, Density Estimation, Manifold Learning and Semi-Supervised Learning [Internet] Microsoft Research 2011 Available from: http://research.microsoft.com/apps/pubs/default.aspx?id=155552

[B20] Mendel J Wu D Perceptual Computing: Aiding People in Making Subjective Judgments 2010 Hoboken, New Jersey: John Wiley & Sons, Inc. 339

[B21] Kinect for Windows SDK from Microsoft Research [Internet]. [cited 2011 Oct 28]. Available from: http://www.microsoft.com/en-us/kinectforwindowsdev/Downloads.aspx

[B22] Calderita LV Bandera JP Bustos P Skiadopoulos A Model-based reinforcement of kinect depth data for human motion capture applications Sensors (Basel) 2013 13 7 8835 8855 10.3390/s130708835 23845933 PMC3758625

[B23] Clark RA Pua Y-H Fortin K Ritchie C Webster KE Denehy L Bryant AL Validity of the Microsoft Kinect for assessment of postural control Gait Posture 2012 36 3 372 377 10.1016/j.gaitpost.2012.03.033 22633015

[B24] Lloréns R Alcañiz M Colomer C Navarro MD Balance recovery through virtual stepping exercises using Kinect skeleton tracking: a follow-up study with chronic stroke patients Stud Health Technol Inform 2012 181 108 112 22954838

[B25] Mobini A Behzadipour S Saadat Foumani M Accuracy of Kinect’s skeleton tracking for upper body rehabilitation applications Disabil Rehabil Assist Technol 2013 [http://dx.doi.org/10.3109/17483107.2013.805825] 10.3109/17483107.2013.805825 23786360

[B26] Khoshelham K Elberink SO Accuracy and resolution of kinect depth data for indoor mapping applications Sensors (Basel) 2012 12 2 1437 1454 22438718 10.3390/s120201437 PMC3304120

[B27] Gabel M Gilad-Bachrach R Renshaw E Schuster A Full body gait analysis with Kinect Conf Proc IEEE Eng Med Biol Soc 2012 2012 1964 1967 23366301 10.1109/EMBC.2012.6346340

[B28] Stone EE Skubic M Capturing habitual, in-home gait parameter trends using an inexpensive depth camera Conf Proc IEEE Eng Med Biol Soc 2012 2012 5106 5109 23367077 10.1109/EMBC.2012.6347142

[B29] Polman CH Reingold SC Banwell B Clanet M Cohen JA Filippi M Fujihara K Havrdova E Hutchinson M Kappos L Lublin FD Montalban X O’Connor P Sandberg-Wollheim M Thompson AJ Waubant E Weinshenker B Wolinsky JS Diagnostic criteria for multiple sclerosis: 2010 revisions to the McDonald criteria Ann Neurol 2011 69 2 292 302 10.1002/ana.22366 21387374 PMC3084507

[B30] Cutter GR Baier ML Rudick RA Cookfair DL Fischer JS Petkau J Syndulko V Weinshenker BG Antel JP Confavreux C Ellison GW Lublin F Miller AE Rao SM Reingold SM Thompson A Willoughby E Development of a multiple sclerosis functional composite as a clinical trial outcome measure Brain 1999 122 Pt 5 871 882 10355672 10.1093/brain/122.5.871

[B31] Socie MJ Sosnoff JJ Gait variability and multiple sclerosis Mult Scler Int 2013 2013 645197 23533759 10.1155/2013/645197 PMC3603667

[B32] Stone EE Skubic M Passive in-home measurement of stride-to-stride gait variability comparing vision and Kinect sensing Conf Proc IEEE Eng Med Biol Soc 2011 2011 6491 6494 22255825 10.1109/IEMBS.2011.6091602

[B33] Crenshaw SJ Royer TD Richards JG Hudson DJ Gait variability in people with multiple sclerosis Mult Scler 2006 12 5 613 619 10.1177/1352458505070609 17086908

[B34] Collongues N Vermersch P Multiple sclerosis spasticity: “state-of-the-art” questionnaire survey of specialized healthcare professionals Expert Rev Neurother 2013 13 3 Suppl 1 21 25 23369056 10.1586/ern.13.10

[B35] Donzé C De Sèze J Spasticity and everyday life in multiple sclerosis Rev Neurol (Paris) 2012 168 3 S51 S56 22721365 10.1016/S0035-3787(12)70047-4

[B36] Phan-Ba R Calay P Grodent P Delrue G Lommers E Delvaux V Motor Fatigue Measurement by Distance-Induced Slow Down of Walking Speed in Multiple Sclerosis PLoS One [Internet]. 2012 Apr 13 [cited 2013 Aug 22];7(4). Available from: http://dx.doi.org/10.1371/journal.pone.0034744 10.1371/journal.pone.0034744 PMC3326046 22514661

[B37] Phan-Ba R Calay P Grodent P Delrue G Lommers E Delvaux V Moonen G Nagels G Belachew S A corrected version of the Timed-25 Foot Walk Test with a dynamic start to capture the maximum ambulation speed in multiple sclerosis patients NeuroRehabilitation 2012 30 4 261 266 22672939 10.3233/NRE-2012-0754

[B38] Fleuren JF Voerman GE Snoek GJ Nene AV Rietman JS Hermens HJ Perception of lower limb spasticity in patients with spinal cord injury Spinal Cord 2009 47 5 396 400 10.1038/sc.2008.153 19065149

